# Prognostic model for aneurysmal subarachnoid hemorrhage patients requiring mechanical ventilation

**DOI:** 10.1002/acn3.51846

**Published:** 2023-07-09

**Authors:** Xichen Wan, Xiao Wu, Junwei Kang, Longjun Fang, Yunliang Tang

**Affiliations:** ^1^ Department of Neurosurgery First Affiliated Hospital of Nanchang University Nanchang 330006 People's Republic of China; ^2^ Department of Rehabilitation Medicine First Affiliated Hospital of Nanchang University Nanchang 330006 People's Republic of China

## Abstract

**Objective:**

Aneurysmal subarachnoid hemorrhage (aSAH) is a major cause of death and disability worldwide and imposes serious burdens on society and individuals. However, predicting the long‐term outcomes in aSAH patients requiring mechanical ventilation remains challenging. We sought to establish a model utilizing the Least Absolute Shrinkage and Selection Operator (LASSO)‐penalized Cox regression to estimate the prognosis of aSAH patients requiring mechanical ventilation, based on regularly utilized and easily accessible clinical variables.

**Methods:**

Data were retrieved from the Dryad Digital Repository. Potentially relevant features were selected using LASSO regression analysis. Multiple Cox proportional hazards analyses were performed to develop a model using the training set. Receiver operating characteristics and calibration curves were used to assess its predictive accuracy and discriminative power. Kaplan–Meier and decision curve analyses (DCA) were used to evaluate the clinical utility of the model.

**Results:**

Independent prognostic factors, including the Simplified Acute Physiology Score 2, early brain injury, rebleeding, and length of intensive care unit stay, were identified and included in the nomogram. In the training set, the area under the curve values for 1‐, 2‐, and 4‐year survival predictions were 0.82, 0.81, and 0.80, respectively. In the validation set, the nomogram exhibited excellent discrimination ability and good calibration. Moreover, DCA demonstrated that the nomogram was clinically beneficial. Finally, a web‐based nomogram was constructed (https://rehablitation.shinyapps.io/aSAH).

**Interpretation:**

Our model is a useful tool for accurately predicting long‐term outcomes in patients with aSAH who require mechanical ventilation and can assist in making individualized interventions by providing valuable information.

## Introduction

Aneurysmal subarachnoid hemorrhage (aSAH), a subtype of hemorrhagic stroke that is associated with high morbidity and mortality, involves the spontaneous rupture of an intracranial aneurysm.^1^, ^2^ It is estimated that over 500,000 people annually develop aSAH,^3^ accounting for over 70% of all forms of SAH.^4^ Despite significant improvements in the clinical treatment of aSAH, patient outcomes remain unsatisfactory.^5^ aSAH has a 45% mortality rate within 30 days, and almost half of those who survive, experience irreversible brain damage from stroke complications, including cephaledema, vasospasm, hydrocephalus, and seizure.^6^ It is crucial to manage patients with aSAH appropriately during the acute phase, prior to aneurysm securement, to improve their survival and functional outcomes.^7^ Research has shown that aSAH patients' outcomes depend on the time required for and the type of aneurysm repair, fluid management methods, and endovascular treatment of delayed cerebral ischemia, among other factors.^8^

Hypoxia can develop because of inadequate oxygenation and ventilation in the brain, which can exacerbate ischemic damage to the brain.^9^ In several studies, low brain oxygenation has been linked to poor neurologic outcomes and mortality following aSAH.^10^, ^11^ An individual with a Glasgow Coma Scale (GCS) score ≤8 is typically incapable of maintaining a patent airway and therefore requires intubation and mechanical ventilation to ensure adequate oxygenation.^12^ It has been reported that 38.5–65% of all aSAH patients require mechanical ventilation.^13^ According to some clinicians, aSAH patients requiring prolonged mechanical ventilation have a poor prognosis.^14^ Therefore, it is important to identify early prognostic predictors for patients with aSAH, as part of their management.^15^ However, the long‐term clinical outcomes of aSAH patients requiring mechanical ventilation remain unpredictable, and no prognostic model has been developed.

Consequently, it is necessary to refine a model for predicting the prognosis of aSAH patients and for guiding clinical treatment. The current study aimed to establish a model utilizing the Least Absolute Shrinkage and Selection Operator (LASSO)‐penalized Cox regression to estimate the prognosis of aSAH patients requiring mechanical ventilation, based on regularly utilized and easily accessible clinical variables. Univariate and multivariate analyses were conducted to identify independent risk variables. A visualization model was created using a nomogram and a web‐based calculator, and the estimated performance was assessed based on discrimination, calibration, and clinical value.

## Methods

### Data source

The datasets analyzed are available from the Dryad Digital Repository (www.datadryad.org), which is an open‐resource database that provides a broad range of discoverable, freely reusable, reference research data. All authors have waived their copyright to these original research data.

### Patients

A secondary retrospective analysis was performed based on a cohort study, which included patients with aSAH hospitalized in the neuro‐intensive care unit (ICU) between January 2010 and December 2015.^16^ Only patients who required mechanical ventilation were included in this study. All patients underwent computed tomography angiography upon admission to confirm subarachnoid hemorrhage caused by aneurysm rupture. Patients with iatrogenic aneurysm ruptures and those lost to follow‐up were excluded from the analysis.

### Data collection

Promising predictive indicators were selected, including age, sex, tobacco use, alcohol abuse, diabetes, cardiovascular disease, anemia localization, GCS score, Simplified Acute Physiology Score 2 (SAPS2), World Federation of Neurological Surgeons Scale (WFNS) score, Fisher's score, intracerebral hemorrhage, early brain injury, external ventricular drain, rebleeding, angiographic vasospasm, delayed cerebral ischemia, length of mechanical ventilation, and length of ICU stay. Furthermore, the vital status and follow‐up duration for each patient with dysphagia were determined.

### Selection of predictive variables and development of the prediction model

To determine the subset of predictors, a univariate Cox regression analysis and LASSO‐penalized Cox regression were performed to select optimal predictors from the risk factors in the training cohort using the “glmnet” package.^17^ Furthermore, based on the results of the LASSO Cox regression analysis,^18^ a multivariate Cox regression model was constructed to predict the outcomes. To enhance its clinical utilization, a web‐based calculator visualization tool was developed.^19^

### Validation of the prediction model

We assessed the predictive ability of our model for survival using the area under the receiver operating characteristic (ROC) curve (AUC) values from the ROC analysis. The training and verification sets were calibrated to evaluate the performance of the novel model.^20^ Decision curve analysis (DCA) was used to evaluate the clinical usefulness of the model.^21^ These assessments were conducted using the training and validation sets.

### Statistical analysis

Normally distributed data are expressed as mean ± standard deviation, whereas non‐normally distributed data are expressed as medians (interquartile ranges). The chi‐square test was used to compare categorical variables between the training and validation sets, whereas the *t*‐test was used to compare continuous variables. Statistical significance was set at *p* < 0.05. Statistical analyses were performed using SPSS (v24.0; IBM SPSS Inc., Armonk, NY, USA) and R (v3.6.2; https://www.r‐project.org/) software.

## Results

### Baseline characteristics and outcomes of subjects

After removing incomplete data, 211 participants were enrolled in this study, of which 60% (*N* = 128) were randomized to the training set and the remaining 40% (*N* = 83) to the validation set (Table S1). The baseline characteristics of each group are presented in Table 1. The median follow‐up period was 638 days. During the follow‐up period, 51 deaths occurred and 77 patients survived in the training dataset, whereas 41 deaths occurred and 42 patients survived in the validation dataset.

**Table 1:** Baseline demographics and clinical characteristics of patients in the training set and validation set.

Variables	All patients (*n* = 211)	Training set (*n* = 128)	Validation set (*n* = 83)	*p*‐value
Age, years	55.46 ± 12.84	55.04 ± 13.19	56.12 ± 12.34	0.551
Gender				0.428
Male	82 (38.9%)	47 (36.7%)	35 (42.2%)	
Female	129 (61.1%)	81 (63.3%)	48 (57.8%)	
Tobacco use				0.083
No	143 (67.8%)	81 (63.3%)	62 (74.7%)	
Yes	68 (32.2%)	47 (36.7%)	21 (25.3%)	
Alcohol abuse				0.796
No	192 (91.0%)	117 (91.4%)	75 (90.4%)	
Yes	19 (9.0%)	11 (8.6%)	8 (9.6%)	
Diabetes				0.249
No	205 (97.2%)	123 (96.1%)	82 (98.8%)	
Yes	6 (2.8%)	5 (3.9%)	1 (1.2%)	
Cardiovascular disease				0.952
No	175 (82.9%)	106 (82.8%)	69 (83.1%)	
Yes	36 (17.1%)	22 (17.2%)	14 (16.9%)	
Anevrism localisation				0.221
Anterior	176 (83.4%)	110 (85.9%)	66 (79.5%)	
Posterior	35 (16.6%)	18 (14.1%)	17 (20.5%)	
Intracerebral hemorrhage				
No	109 (51.7%)	67 (52.3%)	42 (50.6%)	0.805
Yes	102 (48.3%)	61 (47.7%)	41 (49.4%)	
Early brain injury				0.024
No	59 (28.0%)	43 (33.6%)	16 (19.3%)	
Yes	152 (72.0%)	85 (66.4%)	67 (80.7%)	
External ventricular drain				0.461
No	70 (33.2%)	40 (31.3%)	30 (36.1%)	
Yes	141 (66.8%)	88 (68.7%)	53 (63.9%)	
Rebleeding				0.674
No	183 (86.7%)	110 (85.9%)	73 (88.0%)	
Yes	28 (13.3%)	18 (14.1%)	10 (12.0%)	
WFNS score, *n* (%)				0.218
III	22 (10.4%)	12 (9.4%)	10 (12.0%)	
IV	84 (39.8%)	57 (44.5%)	27 (32.5%)	
V	105 (49.8%)	59 (46.1%)	46 (55.4%)	
Fisher score, *n* (%)				0.465
I	1 (0.5%)	0 (0.0%)	1 (1.2%)	
II	4 (1.9%)	3 (2.3%)	1 (1.2%)	
III	40 (18.9%)	22 (17.2%)	18 (21.7%)	
IV	166 (78.7%)	103 (80.5%)	63 (76.0%)	
Angiographic vasospasm				0.743
No	137 (64.9%)	82 (64.1%)	55 (66.3%)	
Yes	74 (35.1%)	46 (35.9%)	28 (33.7%)	
Delayed cerebral Ischemia				0.894
No	154 (73.0%)	93 (72.7%)	61 (73.5%)	
Yes	57 (27.0%)	35 (27.3%)	22 (26.5%)	
Length of mechanical ventilation, days	18.56 ± 17.40	19.52 ± 17.85	17.10 ± 16.68	0.325
Length of stay, days	25.48 ± 23.47	26.66 ± 23.94	23.66 ± 22.74	0.365

WFNS, World Federation of Neurological Surgeons Scale.

### Selection and design of prognostic predictors

Univariate analysis was performed on the training set to identify factors associated with aSAH prognosis. Five valuable factors (SAPS2, early brain injury, rebleeding, length of mechanical ventilation days, and length of ICU stay) were used as prognostic factors for poor outcomes (*p* < 0.05; Table 2). The five clinical features were then entered into the LASSO regression for 1000 bootstrap iterations, and four features with non‐zero coefficients and a minimum lambda value were selected (Fig. S1). According to the multivariate Cox regression analysis, SAPS2, early brain injury, rebleeding, and length of ICU stay were independent prognostic factors (Table 2).

**Table 2:** Univariate and multivariable Cox hazards analysis to screen the potential prognostic factors based on the training cohort.

Variables	Univariate		Multivariate	
	HR (95% CI)	*p*‐value	HR (95% CI)	*p*‐value
Age, years	1.02 (0.99–1.04)	0.161		
Gender	1.42 (0.82–2.48)	0.215		
Tobacco use	0.67 (0.37–1.23)	0.201		
Alcohol abuse	0.98 (0.35–2.72)	0.969		
Diabetes	0.38 (0.05–2.78)	0.343		
Cardiovascular disease	1.07 (0.54–2.14)	0.841		
Anevrism localization	1.19 (0.56–2.53)	0.657		
GCS	0.95 (0.88–1.04)	0.299		
SAPS II	1.03 (1.00–1.05)	0.026	1.04 (1.02–1.07)	0.001
WFNS	1.36 (0.87–2.13)	0.181		
Fisher	1.00 (0.55–1.83)	0.990		
Intracerebral hemorrhage	0.86 (0.50–1.49)	0.594		
Early brain injury	3.04 (1.48–6.25)	0.003	3.13 (1.47–6.63)	0.003
External ventricular drain	0.92 (0.51–1.66)	0.770		
Rebleeding	2.93 (1.52–5.62)	0.001	2.55 (1.28–5.08)	0.008
Angiographic vasospasm	0.62 (0.34–1.16)	0.134		
Delayed cerebral ischemia	1.01 (0.55–1.87)	0.971		
Length of mechanical ventilation	0.98 (0.96–1.00)	0.016		
Length of stay	0.97 (0.96–0.99)	0.002	0.96 (0.95–0.98)	0.000

GCS, Glasgow Coma Scale; SAPS II, Simplified Acute Physiology Score 2; WFNS, World Federation of Neurological Surgeons Scale.

### Development of multivariate prognostic nomogram

**Figure 1:**
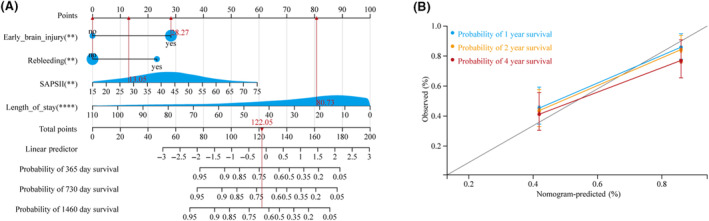
Nomogram of clinical features for predicting survival of aneurysmal subarachnoid hemorrhage (aSAH) patients requiring mechanical ventilation. (A) Nomogram constructed to predict the 1‐, 2‐, and 4‐year survival in the training cohort. (B) Calibration curves for predicting overall survival rate by the nomogram in the training and validation sets.

A multivariate Cox regression model was used to develop a nomogram to discriminate survival (Fig. 1A). A straight line was drawn upward corresponding to each predictor axis, whereas a straight line denoted the total survival axis. Additionally, this prognostic nomogram exhibited good calibration based on the calibration curve (Fig. 1B).

**Figure 2:**
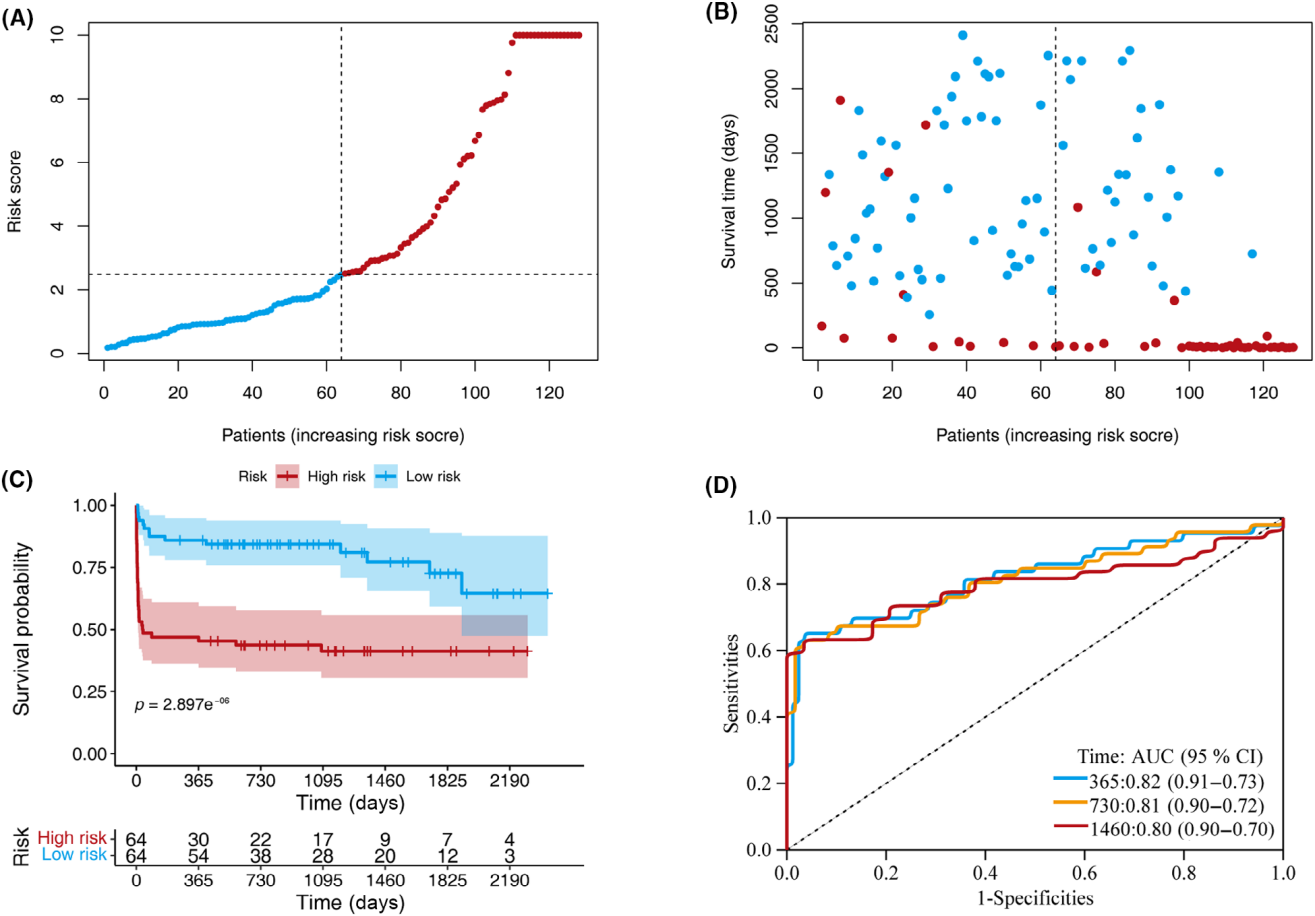
Model discrimination and performance in the training set. (A) Distribution of participants in the low‐ and high‐risk score categories based on survival status. (B) Comparison of survival risk between the two groups. (C) Overall survival curves were separated by groups with low‐ and high‐risk scores. (D) Time‐dependent receiver operating characteristic curves of the scoring system in the training set.

Following the development of this classification model, we stratified 128 trained patients into low‐ and high‐risk groups of 64 patients each. Figure 2A,B show the distribution of the risk scores and survival statuses ranked by the risk scores in the training set. Survival analysis revealed that the high‐risk group had a significantly lower survival rate than the low‐risk group (Fig. 2C). The AUCs for the 1‐, 2‐, and 4‐year survival predictions were 0.82, 0.81, and 0.80, respectively (Fig. 2D), indicating that the model was effective in predicting survival. The nomogram was thus capable of providing valuable and informed prognostic judgment based on DCA (Fig. S2).

### Performance of the model in the validation set

**Figure 3:**
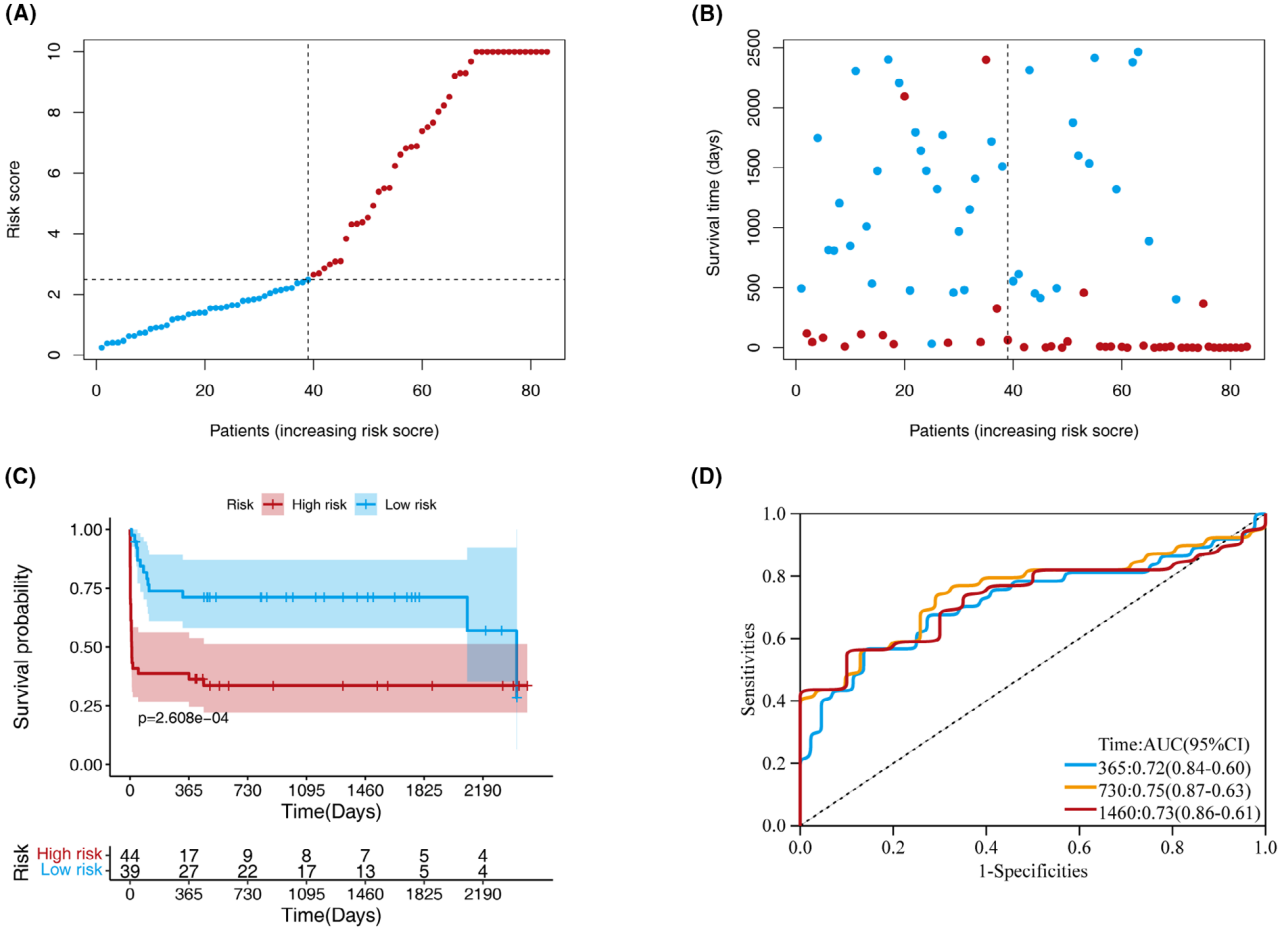
Model discrimination and performance in the validation set. (A) Distribution of participants in the low‐ and high‐risk score categories based on survival status. (B) Comparison of survival risk between the two groups. (C) Overall survival curves were separated by groups with low‐ and high‐risk scores. (D) Time‐dependent receiver operating characteristic curves of the scoring system in the validation set.

A cutoff value was used in the training set to stratify the patients into low‐ (*n* = 39) and high‐risk (*n* = 44) groups in the validation set. Figure 3A,B show the distribution of the risk scores and the survival status ranked by the risk scores in the validation set. Survival analysis revealed that the high‐risk group exhibited significantly worse survival than did the low‐risk group (Fig. 3C). The AUCs for the 1‐, 2‐, and 4‐year survival predictions were 0.72, 0.75, and 0.73, respectively (Fig. 3D).

### Establishment of a web‐based calculator

**Figure 4:**
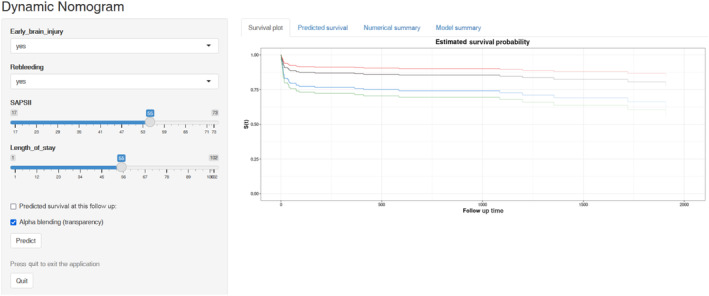
Web‐based dynamic nomogram for prediction of all‐cause mortality probability (https://rehablitation.shinyapps.io/aSAH/). By entering the specific information of the patients with aneurysmal subarachnoid hemorrhage (aSAH) in the web‐based online tool, we could obtain the corresponding all‐cause mortality probability for an aSAH patient requiring mechanical ventilation.

A web‐based prognostic model was developed for easy clinical use and visualization (https://rehablitation.shinyapps.io/aSAH/) of predicted aSAH prognosis based on the established nomogram (Fig. 4). A participant's survival probability can be estimated by entering the details of the aSAH participant into this web‐based program.

## Discussion

SAH is a major cause of death and disability worldwide.^22^ The poor outcome of aSAH can be attributed to the initial catastrophic event or to numerous acute or delayed neurological complications, such as cerebral ischemia, hydrocephalus, or rebleeding.^23^, ^24^ No reliable biomarkers exist for predicting complications and prognoses at clinically relevant timepoints that would allow identification of patients who need aggressive treatment or for determining when a neurological injury can be effectively treated and managed.^25^, ^26^ Recently, a growing number of mathematical models, based on multiple markers, have been developed in medicine using analytical approaches.^27^ This approach involves combining a series of significant parameters to construct a model that performs better in terms of predicting prognosis.^28^ In the present study, clinical data and demographic information of individuals with aSAH were used to estimate the prognostic risk using LASSO‐Cox regression analysis. The SAPS2, early brain injury, rebleeding, and the length of ICU stay were included in the multivariate model. Model performance was validated using discrimination and calibration curves, and DCA in the validation set. The model demonstrated a high degree of predictive power in both the training and validation sets. Furthermore, clinical decision‐making was facilitated with the help of a novel nomogram and a corresponding web‐based calculator. Unlike previous nomograms that calculated estimates, our dynamic nomogram produces exact results.

The predictive power of the model was also evaluated. The predictive validity of the model was assessed by dividing the raw data into training and testing sets. Kaplan–Meier analysis revealed that the high‐ and low‐risk groups had significantly different prognoses. For the training set, the AUCs for 1‐, 2‐, and 4‐year survival predictions were 0.82, 0.81, and 0.80, respectively, while for the validation set, these AUCs were 0.72, 0.75, and 0.73, respectively. In addition, a calibration curve and DCA were used to evaluate the model. The calibration curve and DCA results demonstrated that the method performed well. However, as no similar previous study exists, the performance of our model could not be compared and evaluated owing to the lack of comparable data.

Several studies have investigated biomarkers and constructed nomograms to predict the outcomes in patients with aSAH. Lu et al. developed a nomogram that predicted the risk of 6‐month unfavorable outcomes (by the modified Rankin score) in older patients with aSAH after endovascular coiling.^29^ Hu et al. developed an online prediction tool based on a random forest model to identify patients at a high risk of delayed cerebral ischemia after aSAH.^30^ Fang et al. reported that elevated D‐dimer levels on admission were associated with short‐ and long‐term mortality.^31^ Wiśniewski et al. found that glucose‐6‐phosphate dehydrogenase and 8‐iso‐prostaglandin F2α are potential predictors of delayed cerebral ischemia after aSAH.^32^ However, most of these previous studies focused mainly on neurological complications, and few studies have established survival models. In the present study, we focused on the prognosis of aSAH cases requiring mechanical ventilation, classified as high‐risk after complete resection, and constructed a nomogram to predict mortality in these treated patients.

We recommend the wide application of this novel scoring model in most hospitals for rapid prediction of the prognosis for aSAH patients, as this system is based on four easily accessible clinical variables. Our scoring model could provide an important reference for clinical management, which would be helpful for the selection of therapeutic regimens and the prediction of prognosis.

Among the independent prognostic factors in our model, we should notice that the length of stay is a confounder. It is generally considered that the less length of stay in the hospital seems to be associated with a better prognosis. However, there may be other scenarios that we could not ignore. Some patients stay less time in the hospital because they are dying, and the alive patients had more length of stay. This situation leads to quite different results. Further, well‐designed clinical trials are needed to overcome all the confounders.

This study had several strengths. First, our data suggested that a model is a good option for effectively predicting the survival of patients with aSAH with high accuracy. Second, the features included in this novel model are easy to obtain. Moreover, we developed an easy‐to‐operate web‐based calculator that would be favorable for promoting the precise prevention and personalized health management of aSAH, to maximize cost‐effectiveness.

## Limitations

This study had a few limitations. First, although the performance of the model was good in both the training and validation sets, a multicenter clinical application is required to evaluate the utility of this model externally. Second, this retrospective analysis was based on an open‐access public database, which may have entailed several inherent selection biases. Third, a limited number of covariates were available for investigation and other novel biomarkers were not available. Therefore, a multicenter validation of the scoring system in a large study population is needed to obtain high‐level evidence for the clinical application of the model.

## Conclusion

This study identified and validated a model incorporating four clinical characteristics (SAPS2, early brain injury, rebleeding, and length of ICU stay) to predict long‐term mortality in patients with aSAH. The model performed well in predicting the survival of patients with aSAH. This clinically useful tool can help to improve the prognostic management of aSAH patients.

## Author Contributions

Wan Xichen and Wu Xiao collected and analyzed the data; Kang Junwei and Fang Longjun analyzed and interpreted the data; Tang Yunliang conceived the study.

## Funding Information

This work was supported by the National Natural Science Foundation of China (Nos. 82202811 and 82201271), the Youth Talent Cultivation Project of First Affiliated Hospital of Nanchang University (No. YFYPY202116), the Natural Science Foundation of Jiangxi Province (No. 20224BAB216033), and the Science and Technology Project of Jiangxi Provincial Health Commission (No. 202210362).

## Conflict of Interest

The authors declare that they have no competing interests.

## Ethics Approval and Consent to Participate

Not applicable.

## Consent for Publication

Not applicable.

## Supporting information


Figure S1

Table S1

## Data Availability

All the data used in this study were obtained from the Dryad Digital Repository database (http://www.datadryad.org/).
