# Supplementary material for: Prognostic model for aneurysmal subarachnoid hemorrhage patients requiring mechanical ventilation
Source: Ann Clin Transl Neurol. 2023 Jul 9;10(9):1569–77. doi: 10.1002/acn3.51846 (PMC10502627; doi:10.1002/acn3.51846)
Supplement: Supplementary file 1 — Figure S1 [file ACN3-10-1569-s001.pdf]

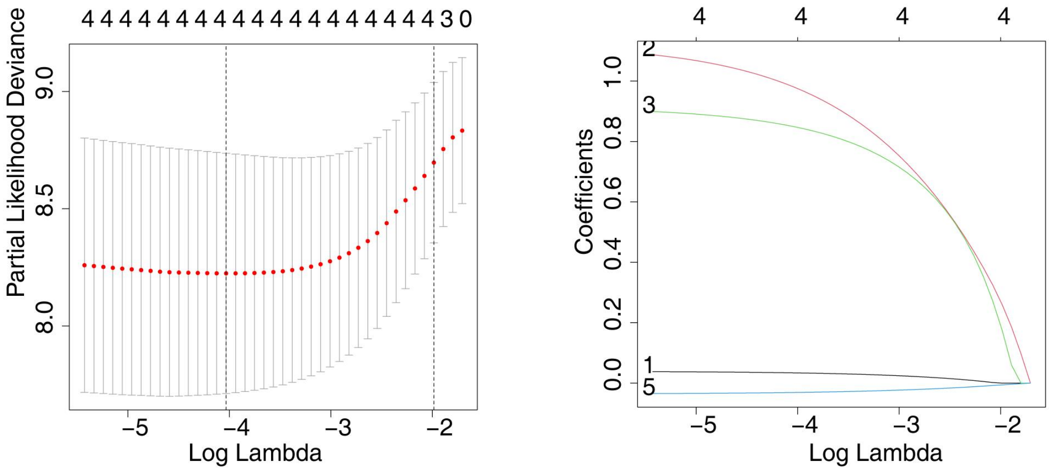

Figure S1: The five clinical features were then entered into the LASSO regression for 1000 bootstrap iterations, and four features with non-zero coefficients and a minimum lambda value were selected.

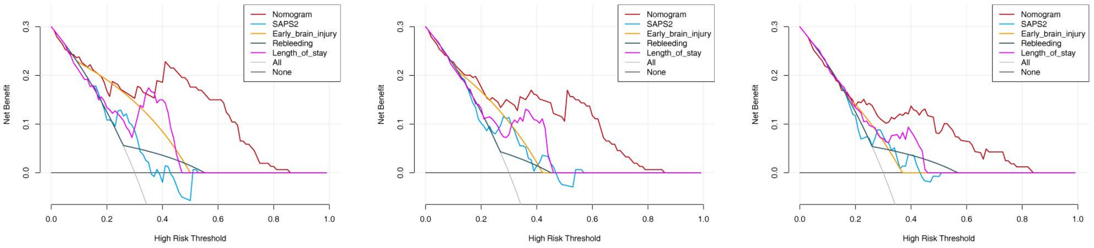

Figure S2: The nomogram was thus capable of providing valuable and informed prognostic judgment based on DCA
